# Operational evaluation of the deployment of Malaria/CRP Duo and Dengue Duo rapid diagnostic tests for the management of febrile illness by village malaria workers in rural Cambodia

**DOI:** 10.1186/s12879-025-11016-z

**Published:** 2025-05-08

**Authors:** Marc T. Visser, Dysoley Lek, Bipin Adhikari, Arjun Chandna, Moul Vanna, Sam Ol, Voeurng Bunreth, Chan Davoeung, Yok Sovann, Dafne Umans, Yoel Lubell, Rusheng Chew, Richard J. Maude, Rob W. van der Pluijm, Rupam Tripura, Naomi Waithira, Siv Sovannaroth, Michèle van Vugt, Lorenz von Seidlein, Mavuto Mukaka, Arjen Dondorp, Thomas J. Peto, James J. Callery

**Affiliations:** 1 Centre of Tropical Medicine and Travel Medicine, Amsterdam University Medical Centres, Location AMC, University of Amsterdam, Amsterdam, The Netherlands; 2 National Centre for Parasitology, Entomology and Malaria Control, Phnom Penh, Cambodia; 3 School of Public Health, National Institute of Public Health, Phnom Penh, Cambodia; 4 Centre for Tropical Medicine and Global Health, Nuffield Department of Clinical Medicine, University of Oxford, Oxford, UK; 5 Mahidol Oxford Tropical Medicine Research Unit, Faculty of Tropical Medicine, Mahidol University, Bangkok, Thailand; 6 Cambodia Oxford Medical Research Unit, Angkor Hospital for Children, Siem Reap, Cambodia; 7 Action for Health Development, Battambang, Cambodia; 8 Battambang Provincial Health Department, Battambang, Cambodia; 9 Pailin Provincial Health Department, Pailin, Cambodia; 10 Amsterdam Institute for Global Health and Development, Amsterdam, The Netherlands; 11 Faculty of Medicine, University of Queensland, Brisbane, Australia; 12 Open University, Milton Keynes, Buckinghamshire, UK; 13 School of Public Health, Li Ka Shing Faculty of Medicine, The University of Hong Kong, Hong Kong, China

**Keywords:** Village malaria worker, Community health worker, RDT, Malaria, Dengue, CRP

## Abstract

**Introduction:**

The decline in malaria cases in Cambodia has led to a relative increase in non-malarial febrile illness. In rural Cambodia, village malaria workers (VMWs) provide early diagnosis and treatment for malaria, but their role and relevance are diminishing as malaria cases decline. Expanding VMW roles would ensure continued utilisation of their services until malaria elimination is achieved and strengthen community health services.

**Methods:**

A mixed methods operational research study was implemented to evaluate the use of two combination-RDTs (combo-RDTs) as an expansion of the VMW role, enabling VMWs in Cambodia to test for diseases other than malaria for the first time. VMWs in 78 villages in Battambang and Pailin Provinces were trained and provided with either a Malaria/CRP Duo or Dengue Duo RDT to assess febrile patients. Data were collected on VMW consultations, and combo-RDT usage and results. Focus group discussions (FGDs) and competency assessments of combo-RDT usage were conducted with VMWs. The main objectives were to determine whether VMWs could perform these combo-RDTs correctly and follow management algorithms, and whether deployment had an impact on VMW consultation rates. Perspectives concerning role expansion and the feasibility of conducting additional tests were also explored.

**Results:**

Between June 2022, and May 2023, a total of 2,425 febrile patients were assessed with either a Malaria/CRP Duo or Dengue Duo RDT. Active dengue infection (NS1- and/or IgM-positive) was identified in 1.2% (11/915) of patients. Positive CRP results (> 20 mg/L) were found for 3.2% (48/1,510) of patients. Following deployment, there was an immediate mean increase of 4.4 VMW consultations per month, from 9.0 to 13.4 (p < 0.01). Competency assessments revealed that some VMWs had difficulty performing the Dengue Duo RDT, particularly in collecting the correct blood volume. This limitation may have led to false-negative dengue NS1 results. VMWs and community members were keen to broaden the skills and responsibilities of VMWs.

**Conclusions:**

Deploying combo-RDTs to VMWs led to a higher utilization of their services. Difficulties performing some aspects of the Dengue Duo RDT, low positivity rates, and a lack of actionable outcomes within the existing context of VMW services suggest that alternative interventions may be better suited for VMW role expansion at this time. Overall, VMWs and community members were receptive to the expansion of the VMW role for a wider range of diseases other than malaria.

**Supplementary Information:**

The online version contains supplementary material available at 10.1186/s12879-025-11016-z.

## Introduction

Malaria cases in Southeast Asia are steadily declining, resulting in the cause of febrile illness increasingly being of non-malarial aetiology [1–4]. In Cambodia, there were only 1,384 malaria cases in 2023, from a recent high of over 63,000 in 2018 [5]. Village malaria workers (VMWs) play a vital role in malaria control and elimination, providing early diagnosis and treatment services in rural areas. VMWs are a specific type of community health worker (CHW) trained exclusively to manage malaria in the community where they live but have limited or no responsibility for managing non-malarial febrile illnesses [6, 7]. With the significant decline in malaria cases, the role, relevance, and social standing of VMWs are diminishing [8, 9]. In western Cambodia, the number of VMW consultations has decreased from 33,800 in 2019 to less than 20,000 in 2023 [5]. VMWs are essential for malaria control in Cambodia [7], and CHWs have been shown to maintain malaria elimination activities during the pre-elimination phase in other low- and middle-income countries [10–14]. Expanding their roles would ensure that febrile patients continue to use their services until local elimination is achieved and the risk of reimportation subsides —a critical strategy to achieve malaria elimination [15]. 

Many countries have implemented CHW programmes for a range of other health issues, such as child nutrition, vaccination campaigns, common childhood illnesses, and directly observed therapy for tuberculosis [16–19], however, how to best expand the roles of VMWs is not yet clear. In Myanmar, expanding the roles of community malaria workers, thereby developing them into CHWs, led to a sustained uptake of their services and a decline in malaria cases [20]. In contrast, two large studies with long-term surveillance in other areas of Myanmar did not observe a reduction in patient attendance despite a significant decline in *P. falciparum* malaria over a seven-year period [21, 22]. 

Cambodia’s National Malaria Control Program (CNMCP) has trained and devolved the role of malaria management to VMWs for the last 20 years [6, 23]. VMWs test for malaria with rapid diagnostic tests (RDTs), interpret them, and provide antimalarials based on results [24]. The relative rise of non-malarial febrile illness combined with the VMWs’ experience with RDTs prompted the idea that using diagnostic tests for non-malarial illness would be a logical step in expanding their role. The aetiology of non-malarial febrile illness in rural Cambodia is not well defined, but studies have demonstrated local transmission of dengue, Japanese encephalitis, leptospirosis, scrub typhus, hepatitis E, and influenza [3, 4, 25, 26]. For some of these pathogens, novel RDTs offer point-of-care testing with results available within minutes [27]. Dengue and C-reactive protein (CRP) RDTs are commercially available and could be used for this purpose. In rural areas with diverse aetiologies of febrile illness, such as Cambodia, empirical treatment with antimicrobials is commonly provided without a definitive diagnosis, and patients often self-treat with over-the-counter medicines [4]. The introduction of non-malarial RDTs could mitigate the unnecessary use of antimicrobials and provide a more accurate diagnosis and appropriate management. These gains would need to be weighed against the use of additional resources.

The incidence of dengue in Cambodia has increased significantly over the last 20 years, with the most recent epidemic in 2019 being the largest on record [28]. During an outbreak of dengue serotype 3 in 2007, rural areas were affected more than urban areas [29]. Despite the underreporting of the disease burden in Cambodia, the costs associated with dengue are high [30, 31]. Non-severe dengue infections require conservative treatment only, i.e., emphasising the importance of managing fever and monitoring for warning signs of severe illness. This makes dengue a suitable disease for initial management by VMWs.

Malaria-negative patients with undifferentiated fever are often treated with antibiotics because of limited access to diagnostics for other pathogens [32, 33]. This behaviour drives inappropriate antibiotic usage, thus promoting antibiotic resistance, a major threat to human health [34]. CRP, a non-specific marker of inflammation, can be indicative of a bacterial infection [35] and has shown potential to steward antibiotic usage in Southeast Asia [36–39]. If testing was available at the village level, this could potentially identify patients with low CRP levels who are unlikely to benefit from antibiotic therapy, circumventing the need for them to travel the often long distances to their nearest health centre. Additionally, within an appropriate governance framework, testing could support the safe and judicious expansion of antibiotic prescribing from healthcare providers in primary health centres to their lesser-trained counterparts in the community, thereby improving universal health coverage, particularly for patients residing in hard-to-reach rural areas.

The main objectives of this innovative study were to determine: (1) whether VMWs in the rural villages of Battambang and Pailin Provinces in western Cambodia could correctly use two new combination RDTs (combo-RDTs)—the Dengue Duo and Malaria/CRP Duo—and appropriately follow their respective management algorithms; and (2) whether the introduction of these combo-RDTs would help slow the decline in the number of consultations with VMWs, thereby supporting malaria elimination efforts. This study represents the first time that VMWs in Cambodia have been given the capability to test for diseases other than malaria. Further objectives included exploring perspectives around expanding VMW roles and the feasibility of VMWs conducting additional tests.

## Materials and methods

### Design and study setting

This mixed methods study was implemented within two provinces (Battambang and Pailin) in western Cambodia as part of an operational research project to evaluate the feasibility of expanding the VMW role. Battambang and Pailin provinces were chosen as they had been identified as areas approaching malaria elimination. VMWs were provided with new combo-RDTs to use during their assessment of febrile patients (an axillary temperature ≥37.5 °C, or a history of fever in the previous 24 h) in addition to their routine malaria testing. Only patients requiring urgent medical attention at the point of presentation were excluded from the study.

**Fig. 1:**
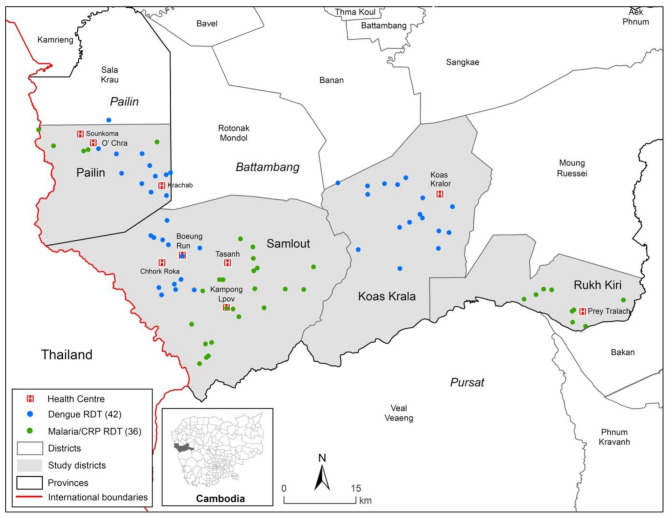
Map of the study area. Location of the study villages and health centres in Battambang and Pailin Provinces in western Cambodia.

This operational research project was undertaken in 78 villages within nine health centre catchment areas in four operational districts (Koas Krala, Pailin, Rukh Kiri, and Samlout), all of which are close to achieving local malaria elimination [Figure 1].

The quantitative aspect of this mixed methods study included two main components: (1) an evaluation of combo-RDTs as an intervention to help slow the decline in consultations with VMWs, and (2) an assessment of VMWs' disease knowledge and their use of combo-RDTs through a competency assessment questionnaire. The qualitative component utilised focus group discussions (FGDs) to explore the experiences and perspectives of VMWs regarding the potential expansion of their role.

### Rapid diagnostic tests

Following consultation with local and national stakeholders, and experts in diagnostic tests, two combo-RDTs were selected for use by VMWs: the STANDARD™ Q Malaria/CRP Duo (SD Biosensor) and the STANDARD™ Q Dengue Duo (SD Biosensor) tests [40, 41]. The reasons for selecting these combo-RDTs included their ease of use, high reference test correlation, compatibility with whole blood sampling, an ability to provide results that were amenable to VMW management in the community, an expectation of obtaining many actionable results, and that these tests met the procurement criteria set by the funder. A dual dengue test was specifically chosen following a regionally conducted study that reported a significant improvement in sensitivity following the addition of a serological component [42]. 

Both combo-RDTs are lateral flow immunochromatographic assays and consist of two tests packaged side by side. The Malaria/CRP Duo malaria test recognises histidine-rich protein 2 (HRP-2) for *Plasmodium falciparum* and plasmodium lactate dehydrogenase (pLDH) for *P. falciparum*,* P. vivax*,* P. ovale* and *P. malariae* (PAN malaria). The cut-off for a positive CRP result is 20 mg/L. This combo-RDT requires 15 µL of whole blood. The Dengue Duo RDT tests for the acute phase non-structural protein 1 (NS1) antigen and IgG and IgM antibodies against dengue virus and requires 110 µL of whole blood. For both combo-RDTs, the manufacturer recommends reading the results after 15 to 20 min.

Malaria/CRP Duo RDTs were provided to all VMWs working within the catchment areas of Tasanh, Kampong Lpov, Prey Tralach, Soun Koma and O’Chra health centres and Dengue Duo RDTs were provided to all VMWs working in Koas Kralor, Boeung Run, Chhork Roka and Krachab. Allocation decisions were pragmatic following discussions with the team implementing the operational aspect of the study. Considerations included the need to provide training and deliver the RDTs over a large geographical area in a timely manner, whilst also balancing the number of villages/VMWs provided with each combo-RDT. During the study period, VMWs were required to test patients with the CNMCP-provided malaria RDT (FalciVax Rapid Test for Malaria Pv/Pf, Zephyr Biomedical Systems) simultaneously, with either the Dengue Duo or Malaria/CRP RDT.

VMWs were provided with a simple management algorithm flowchart to determine which febrile patients should be referred to a primary health centre [Appendix I]. Although in the future it may be feasible for VMWs to manage uncomplicated patients with positive Dengue Duo or Malaria/CRP results, in the context of this operational research study, which was primarily designed to assess the feasibility of expanding the VMW point-of-care testing repertoire, VMWs were recommended to refer all patients with positive CRP or dengue NS1/IgM results to their nearest health centre for further assessment. Nevertheless, VMWs and patients were ultimately free to make their own management decisions. Malaria cases were managed according to national guidelines in line with existing VMW practice [43]. Patients with a negative combo-RDT result were advised on simple fever management and to present for reassessment after 48 h if symptoms persisted. The algorithm was developed by the study team in collaboration with the Pailin and Battambang Provincial Health Departments.

### Process indicators

Data on VMW consultations, combo-RDT usage and results, and patient characteristics were entered by the VMWs into an electronic case record form (eCRF) developed using the Commcare platform (Dimagi Inc., Cambridge, MA, USA) on Android tablets. Routine VMW malaria testing data from prior to and during combo-RDT deployment were obtained from the registry available at the health centres. Corresponding data for the whole of Battambang and Pailin Provinces were obtained from the CNMCP.

### Competency assessment

Before the rollout, VMWs were trained to use the combo-RDTs during monthly VMW meetings at health centres. Training consisted of a full day of hands-on practice using the allocated combo-RDT whilst also familiarising themselves with the management algorithm flowchart, the eCRF, and other study processes. Training was delivered at each of the nine health centres separately. VMWs informed their communities of the additional services and began using the combo-RDTs immediately following training. Regular monitoring of the VMWs was undertaken by the operational study team and additional training was provided if required. A competency assessment questionnaire was developed to assess the level of skills acquired and retained by VMWs [Appendix II]. Based on their willingness to participate and limited by logistical feasibility, 16 VMWs were selected for competency assessment. These assessments were conducted during routine visits to VMWs by the field team or at monthly VMW meetings, approximately five months after combo-RDT deployment. The assessment consisted of (1) questions regarding disease symptoms and their infectious nature, (2) the performance of the combo-RDT in question, (3) the interpretation of test results, and (4) whether management algorithms were appropriately followed.

### Perspectives on VMW role expansion

To explore the experiences and perspectives of VMWs related to the potential expansion of their roles, specifically the use of combo-RDTs, focus group discussions (FGDs) were held. All VMWs attending a regular monthly supervision meeting were invited to join a FGD on either the Malaria/CRP Duo or Dengue Duo RDT, depending on which they had been allocated.

A FGD guide was prepared in advance to respond to the research questions of the study. The FGD guide aimed to explore the perspectives around expanding the VMW role, managing non-malarial illnesses in the community, and the feasibility of conducting combo-RDTs within the current VMW role, specifically the ease of use, interpretation of results, relevance, benefits, and drawbacks. All FGDs were audio-recorded after written informed consent was obtained from the participants and later transcribed and professionally translated from Khmer to English. Khmer researchers checked and verified the accuracy of the transcriptions before analysis.

To obtain the perspectives of other key stakeholders, such as health centre workers and village chiefs and residents, FGDs were supplemented with notes from participant observations. These observations were made and recorded by research staff during their visits to the study area to assist with the operational aspects of implementing the study. Stakeholders were selected purposively based on their experience and exposure to the VMWs conducting the additional tests, in combination with their willingness to participate. Field notes were written in Khmer, translated into English, and accuracy confirmed with the note taker.

### Data analysis

#### Rapid diagnostic tests

An interrupted time series analysis was conducted to evaluate the impact of the deployment of combo-RDTs on the number of consultations with VMWs over time. Since time series observations are often correlated over time (autocorrelated), the analysis utilized the Prais-Winsten method to account for this. The deployment of combo-RDTs was anticipated to have an immediate effect on the number of consultations, so a slope and level change Prais-Winsten regression model was constructed. This model estimates both the immediate change and the change in time trend associated with the deployment of combo-RDTs using the *prais* R package (v1.1.2). The estimates, along with their 95% confidence intervals, have been reported. Statistical significance was declared at the 5% level. As part of a sensitivity analysis, alternate models were fitted that included: (1) an adjustment for seasonality using a categorical month indicator and (2) an adjustment for seasonality using a categorical wet season indicator [Appendix III]. Combo-RDT usage and results were analysed using descriptive statistics, presenting both absolute (n) and relative frequencies (%). Statistical analyses were performed using R Statistical software, version 4.4.1.

#### VMW role expansion

FGD transcriptions and observation notes were collated and coded line by line in Microsoft Word. All coded transcripts were organised in Microsoft Excel to explore their patterns and prominence. Based on the research question and their relevance, codes were coalesced to build minor and major themes. These themes were discussed with the study team and revised based on the feedback from their interpretation and the relevance to the research question.

## Results

All VMWs active in the study area (*n* = 84) were recruited, trained and supplied with combo-RDTs, tablets loaded with the Commcare application installed, and management algorithms to support the assessment of febrile patients presenting in the rural communities of Battambang and Pailin Provinces from June 2022 to May 2023. During this one-year period, a total of 2,425 febrile patients were assessed with a combo-RDT (Dengue Duo, *n* = 915; Malaria/CRP, *n* = 1,510) [Table 1]. Patients’ ages ranged from 7 months to 86 years old (median 32 years, IQR 17–48). Active dengue infection (NS1- and/or IgM-positive) was identified in 11 out of 915 patients (1.2%). Malaria/CRP RDT testing resulted in 48 out of 1,510 (3.2%) patients testing CRP-positive (> 20 mg/L), and three *P. falciparum*-positive results being recorded on the eCRF. Routine CNMCP malaria testing conducted alongside this study confirmed that two of these results were negative whilst the third did not undergo duplicate testing. No cases of *P. falciparum* were officially recorded or treated by the CNMCP in the study area during this time, suggesting that any instances recorded on the study eCRF were documented in error. Among those with a positive CRP result, VMWs advised a referral in 73% (35/48) of cases, with 43% (15/35) of those attending a healthcare facility. Antibiotic treatment was prescribed for 87% (13/15) of healthcare facility attendees. Two (2/11;18%) dengue-positive (NS1- and/or IgM-positive) patients and three patients who were positive for IgG alone were recommended to attend a healthcare facility for confirmation of diagnosis and further management. None of these required onward referrals to a hospital following attendance at a health centre. The remaining nine (9/11; 82%) dengue-positive (NS1- and/or IgM-positive) patients were provided with fever management and safety netting advice instead of being referred to a health centre. As a cross-sectional operational research study, clinical outcomes beyond the day of enrolment were not routinely collected. Informal reporting from community members to the operational field team revealed that there were no deaths or reports of adverse outcomes/severe illness resulting in hospitalisation during the study.

**Table 1:** Febrile patient characteristics and combo-RDT results and referrals

**Febrile patient characteristics**	*n* = 2425
Sex (Male)	1103 (46%)
Age (years), median (IQR)	32 (17 to 48)
Children (< 5 years)	59 (2%)
**Combo-RDT allocation**	
Dengue Duo	915 (38%)
Malaria/CRP Duo	1510 (62%)
**Combo-RDT results**	
**Dengue Duo**	*n* = 915
Positive cases (NS1- and/or IgM-positive)	11 (1.2%)
NS1 + IgM	1 (0.1%)
IgM + IgG (NS1-negative)	10 (1.1%)
IgG only (NS1-negative)	8 (0.9%)
**Malaria/CRP Duo**	*n* = 1510^a^
Malaria	
*P. falciparum*	3 (0.2%)^b^
*P. vivax/ovale/malariae* (PAN)	0 (0.0%)
CRP	
Positive (> 20 mg/L)	48 (3.2%)
**Positive tests referred to a health centre**	
**Dengue Duo**	
Positive cases (NS1- or IgM-positive)	2/11 (18.2%)
IgG only	3/8 (37.5%)
**Malaria/CRP Duo**	
CRP positive (> 20 mg/L)	35/48 (72.9%)

^a^ Some patients had only one of the tests done from the Malaria/CRP Duo (malaria only, *n* = 74; CRP only, *n* = 2)

^b^ Cambodian national malaria control programme (CNMCP) testing confirmed that two of these results were negative. The third did not undergo duplicate testing. No cases of *P. falciparum* were officially recorded/treated by the CNMCP in the study area during this time

Note: RDTs were repeated for some patients if a test was invalid or further confirmation was needed (Dengue Duo, *n* = 33; Malaria/CRP, *n* = 80). To avoid duplicate results, only the second, repeated result was used in the analysis

### VMW consultations

**Fig. 2:**
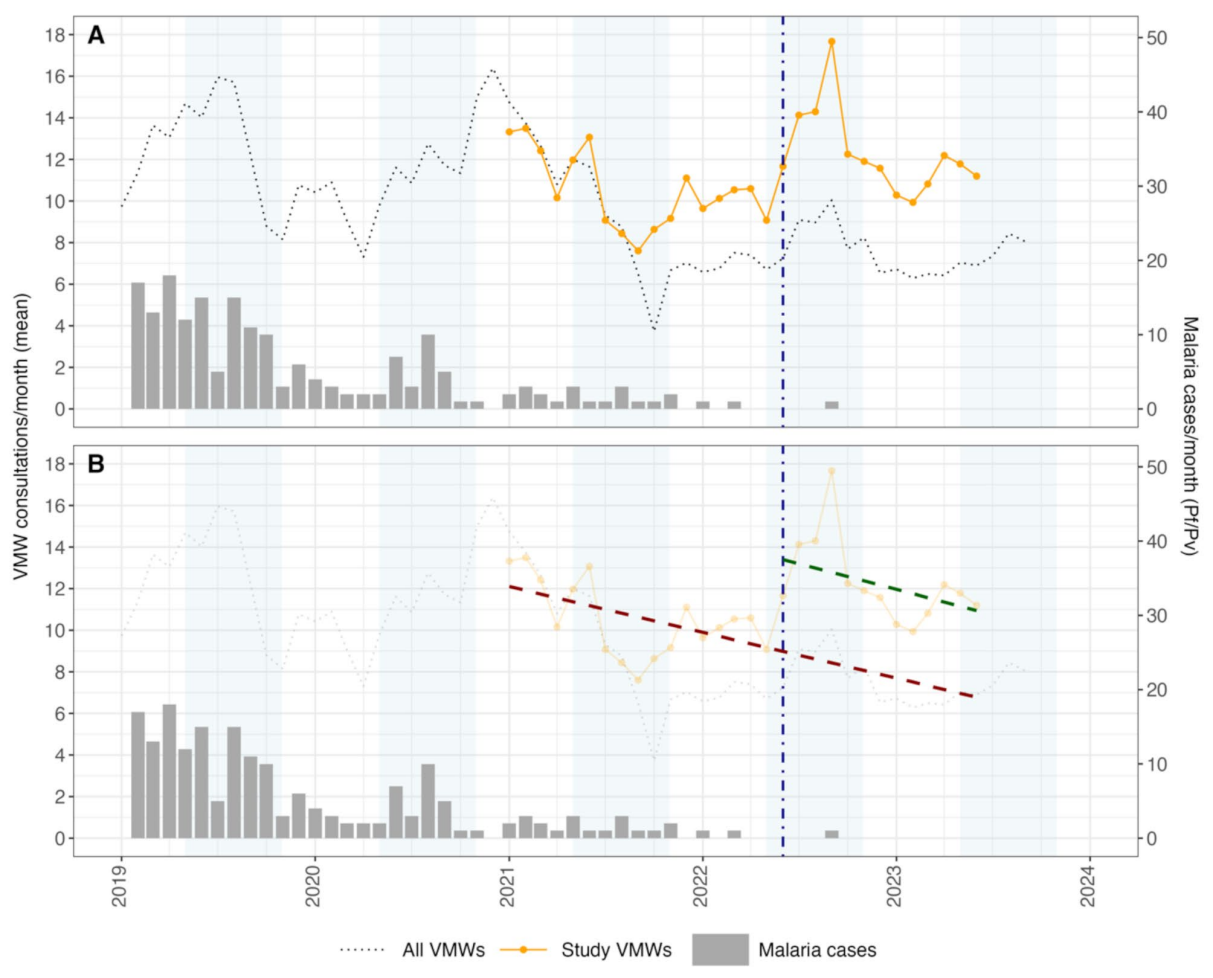
VMW consultation rates and malaria cases in Battambang and Pailin Provinces from 2019 to 2023. ***(A)*** *Mean monthly VMW consultations for the study VMWs (orange line) and all VMWs in Battambang/Pailin Provinces (black dotted line). The vertical blue line denotes the time when the Dengue Duo and Malaria/CRP RDTs (combo-RDTs) were deployed. The bar chart depicts the total monthly malaria cases (Pf/Pv) in Battambang/Pailin Provinces. The blue panels represent the timing of the rainy season.****(B)*** *As per panel A with trend lines for the study VMWs overlayed. Observed and predicted pre-intervention trend line (red dashed line) and observed post-intervention trend line (green dashed line)*

During the implementation period, 10,072 consultations occurred with the 84 VMWs. In the 3.5 years prior to combo-RDT deployment, there was a decline in the number of consultations with VMWs across the whole of Battambang and Pailin Provinces from 9.7 to 6.7 consultations per month (trend: -0.12 consultations per month, 95% CI -0.24 to 0.01; *p* = 0.07) [Table 2; Fig. 2 and Supplementary appendix V]. Data collected in the study area in the 18 months prior to combo-RDT deployment demonstrated a similar rate of decline from a mean of 13.3 to 9.1 consultations per month (trend: -0.18 consultations per month, 95% CI -0.40 to 0.04; *p* = 0.11). Following the deployment of the combo-RDTs, there was an immediate step-change increase, leading to an additional 4.4 consultations per month (95% CI 1.5 to 7.4; p < 0.01). The trend for the mean number of month-to-month VMW consultations, although highly uncertain, showed a slight decline over the subsequent 13 months of combo-RDT usage (trend: -0.02 consultations per month, 95% CI -0.43 to 0.39; *p* = 0.92). Results of the sensitivity analysis were similar when adjusting for seasonality with (1) a categorical month indicator and (2) a categorical wet season indicator (step-change: 4.7 and 4.5; post-intervention trend: -0.02 and − 0.04, respectively) [Supplementary appendix III].

**Table 2:** Interrupted time series analysis: VMW consultations before and after RDT deployment using Prais-Winsten regression

	Pre-intervention trend ^a^ (consultations per month), (95% CI); *p* value	Step change at RDT introduction (consultations per month), 95% CI); *p* value	Post-intervention trend (consultations per month), 95% CI); *p* value
Study VMWs (combo-RDTs) $\:n$ *= 84*	-0.18 (-0.40 to 0.04); *p* = 0.11	4.4 (1.5 to 7.4); p < 0.01	-0.02 (-0.43 to 0.39); *p* = 0.92
All VMWs $\overline n$ *= 152*	-0.12 (-0.24 to 0.01); *p* = 0.07	*N/A*	*N/A*

^a^ Pre-intervention trend in the study area was derived from VMW data collected in the 18 months prior to deployment.

Pre-intervention trend for “All VMWs” was derived from routine national VMW data collected in the 3 ½ years prior deployment

*p*-values refer to a null effect, i.e., trend: slope = 0 and step-change = 0, $\overline n$ is the mean number of VMWs over the 3 ½ years

Study VMWs: ρ = 0.38, Durbin-Watson = 1.88

Adjusting for seasonality with a categorical month indicator and a categorical wet season indicator did not improve the model

VMWs– Village malaria workers, CI– confidence intervals, N/A– Not applicable

### Competency assessment

Competency and disease knowledge were assessed for 16 VMWs (Malaria/CRP Duo RDT, *n* = 8; Dengue Duo RDT, *n* = 8) from five health centre catchment areas (Prey Tralach, Tasanh, Krachab, Koas Kralor, Boeung Run) [Appendices II and IV].

### Malaria/CRP

Basic disease knowledge for malaria was excellent (100% correct), but CRP knowledge was lacking, with 50% unaware that a raised CRP value indicates a higher probability that an infection is of bacterial origin. Practical performance was excellent for the malaria test (100% correct) but less so for the CRP test (63% correct). Two VMWs (25%) used a 100 µL pipet to collect blood for CRP testing when only 10 µL was needed. Test result interpretation (using example images) and suggested management were performed without error.

### Dengue duo

All the VMWs answered basic disease knowledge questions correctly. The practical performance of the NS1 test demonstrated that six of eight VMWs (75%) had difficulties obtaining the required 100 µL of blood, ultimately resulting in one invalid test. The antibody test (IgG/IgM) was performed correctly seven times (88%), while one VMW (13%) had the materials confused and deposited the blood meant for the antibody test in the NS1 test device, also using the wrong pipet. Six VMWs (75%) had no trouble interpreting test results and suggesting management. A faint line was interpreted as invalid instead of positive by one VMW, and one was confused with the difference between IgG and IgM.

### Perspectives on VMW role expansion

Four FGDs were conducted among a total of 29 participants, and each FGD consisted of six to nine participants [Table 3]. From the thematic analysis of the FGDs and field notes, three main themes relevant to the current research question were identified: (1) motivation for role expansion, (2) patient management, and (3) practical concerns.

Despite the low prevalence of malaria in the region, the need for continued malaria testing was deemed essential among the VMWs and their communities. Most VMWs responded with a positive attitude toward the new tests. Among the list of diseases, tests for tuberculosis, typhoid fever and diabetes were requested, as the perceived burden of these diseases was considered high within their communities.

**Table 3:** Focus group discussion: VMW participant characteristics

Characteristic	n = 29
Gender, n (%)	
Male	10 (34%)
Female	19 (66%)
Age (years), median (IQR)	45 (37 to 57)
Years of experience as a VWM, median (IQR)	11 (7 to 18)
FGDs held	
Malaria/CRP Duo	2
Dengue Duo	2
Group size, range	
Malaria/CRP Duo	6–7
Dengue Duo	7–9

Focus group discussions on the Malaria/CRP Duo RDT were held with VMWs from Prey Tralach, O’Chra and Soun, and on the Dengue Duo RDT with VMWs from Krachab and Chhork Roka

### Motivations for role expansion

VMWs’ motivations for their role expansion were embedded broadly into two main interconnected objectives: (1) to improve the overall quality and access to community-based health services altruistically, which in turn, (2) enhanced their standing, trust, and relationships with the community, supported by their newly gained knowledge and skills. Expanding the role of VMWs translated into serving patients in the community in multiple ways. Point-of-care dengue testing improved the VMW’s ability to reassure dengue-negative febrile patients, and new knowledge enabled them to provide health-related messages to community members confidently.

It’s important to help people with fever and to reduce dengue fever so that patients seek an appropriate health service. We are more accessible to them.

VMW using the Dengue Duo RDT at Krachab HC during a FGD with nine participants.

The distance to the health centre, means of transport, direct and indirect travel expenses, and the limited availability of health services were all major barriers to community members accessing healthcare. Being aware of these barriers, VMWs realised that expanding their roles could help alleviate the disease burden by mitigating expenses that a private clinic would charge patients.

The communities can reduce expense or time on going to get a blood test at the centre. […], it reduces the expense of going [to get blood test] at private [clinics]. That’s what I have from my thoughts.

VMW using the Dengue Duo RDT at Chhork Roka HC during a FGD with seven participants.

### Patient management

In their primary role as malaria testers, VMWs were comfortable giving advice regarding fever. They could treat falciparum malaria but referred *P. vivax*-positive patients to health centres for G6PD testing and treatment (as recommended by the CNMCP). VMWs shared the limitations of their roles and skills. For example, they were concerned that they could only diagnose dengue but not provide further management during the current study, spontaneously contrasting their roles and competence with how they manage malaria cases, for which they can confidently diagnose and immediately treat following years of experience and training. Yet, they acknowledged the importance of dengue-negative results as dengue is perceived as a frequent cause of fever in their communities.

*“What should you do when the result is negative?”* [Interviewer]

With a negative result, we can only inform them that they do not suffer from dengue or malaria. We are not able to diagnose other diseases for them, and even if they suffer from other diseases, we do not have medicines to cure them because we just follow the guidelines, and there is no training on how to cure other diseases. We do not have medicines for dengue on hand, only for malaria.

VMW using the Dengue Duo RDT at Chhork Roka HC during a FGD with seven participants.

VMWs were aware of the potential adversities arising from their practice in the community. VMWs shared how potential discrepancies in the test outcomes between themselves and the private clinics could affect their perceived competence and trust by community members.

So, what I would like to confirm is that, if our result is negative, but that of the private clinic is positive, like we discussed just now, how reliable is our test for the people in this society?

They no longer trust me because the result with my test is negative, but the result at the private clinic is positive, and they recovered from their treatment. I think they no longer trust us but trust the private clinic.

VMWs using the Dengue Duo RDT at Chhork Roka HC during a FGD with seven participants.

In rural villages, patients often visit private clinics for verification and definitive diagnosis. VMWs were acutely aware of instances of poor practice occurring at private clinics, where patients were often misled or over-treated. This created a tension that threatened the trust between VMWs and their communities. VMWs described that, owing to potential conflicts of interest, private clinics may attempt to invalidate the results coming from VMWs to fulfil their business interests, e.g., interpreting an IgG-positive dengue result (indicative of previous exposure) as an active dengue infection requiring some form of treatment. Sometimes, overtreatment of patients with saline infusion as a means to cure an often self-limiting disease such as dengue was reported to contradict the suggestions of VMWs, thus compromising the trust relationship with VMWs.

If that happened, what would you think if we took the blood test and the result showed negative? And then they approached a private clinic, and the result was positive?

They no longer trusted our test. It was possibly their way of doing business. We had no idea about that. It’s impossible to inject a serum to heal dengue fever. People in the community don’t know about it. They would get any medicine from a private clinic. We did not know. They did not let us see it. They just took the blood and kept it at their place. Then, the clinic told the patient that they had dengue fever. They then injected a serum. They never showed the result to the patient. They only told them about the result.

VMWs using the Dengue Duo RDT at Krachab HC during a FGD with nine participants.

When discussing the need to refer positive RDT results, VMWs stated that patients were sometimes dismissive of a referral to a government health centre, preferring instead to visit private practices or hospitals. Factors impeding health centre referrals included a limited means of transportation, distance, cost, and a lack of trust in the formal healthcare system.

VMWs showed adequate confidence when diagnosing a patient with malaria, with a complete package ranging from providing treatment to preventative measures at home, along with maintaining the hygiene of themselves and the environment.

For malaria, if the patients come to us, we will do a blood test for them to diagnose their symptoms. If the result is positive with a minor disease, we will cure them; but if the result is negative, we will advise them to keep their body hygienic by bathing, cleaning, and sleeping in medicated mosquito nets. We also advise them to keep the environment around their house clean. If the test result is positive for other diseases, we do not provide treatment to them, but we advise them to go to the health centre for treatment and medication.

VMW using the Malaria/CRP Duo RDT at Prey Tralach HC during a FGD with six participants.

### Practical concerns

While most VMWs stated that they were content with the training they received, some reported difficulties and were open to more extensive practical training. The ability to obtain 100 µL of blood via finger prick for the dengue NS1 test was considered too large of a volume, and the use of multiple different-sized pipettes (100 µL and 10 µL) was also confusing for some.

There should be a reduction in the bigger one [100 µL pipette] because it is hard to be used for pumping blood as the volume of its air is big, and if we incidentally release [our fingers] from squeezing it, we will not get any blood out. This means this one is hard to be used, whilst the smaller one is the most convenient. The smaller tube requires less blood, and compared with the bigger one, it is just a little smaller.

VMW using the Dengue Duo RDT at Chhork Roka HC during a FGD with seven participants.

It’s a little complicated. If you take blood once by using one tube, you would not need to change it. This means that you put it in one tube and divide the liquid into two?

VMW using the Dengue Duo RDT at Krachab HC during a FGD with nine participants.

Furthermore, the Dengue Duo RDT yielded multiple results at once. Possible combinations of positive/negative NS1, IgG and IgM results were at times confusing. Nonetheless, the VMWs were able to interpret the outcomes and praised the speed at which results were available for patients.

VMWs provided with the Malaria/CRP Duo RDT were satisfied with the ease of its use. The malaria test was similar to the one provided by the CNMCP; it followed the same steps and also required 5 µL of blood. The additional 10 µL required for the CRP test did not pose a barrier. When asked if adding multiple point-of-care tests would be feasible, VMWs were unsure, as they feared that the need for extra blood may be unpopular with patients in their communities.

## Discussion

Expanding the roles of VMWs is one potential solution to ensure the continued uptake of their malaria services until malaria elimination has been achieved and the risk of reimportation subsides. It may also increase the reach of primary healthcare services beyond this time. This study evaluated the use of two combo-RDTs by VMWs in rural Cambodia and explored the perspectives around expanded VMW roles and the feasibility and practicalities of VMWs conducting additional tests. This represents the first time that VMWs in Cambodia have gained the ability to diagnose diseases other than malaria with RDTs.

### Non-malarial RDT usage

The frequency of positive tests (CRP > 20 mg/L; Dengue NS1- and/or IgM-positive) with the new combo-RDTs was low (1.2–3.2%), which raises the question of whether the selected combo-RDTs aligned with the epidemiological characteristics of the region, however, negative results and the actions taken were also important and useful outcomes. Despite this, the mean number of consultations with a VMW increased by an additional 4.4 consultations per VMW per month following combo-RDT deployment. Nevertheless, other study activities such as regular monthly meetings with VMWs (conducted quarterly prior to study implementation), support and additional visits to the villages from research staff, and VMW health education may have also contributed to the observed changes [9, 44]. As expected, few malaria cases were found, yet VMWs and community members were aware of the need for continued malaria testing. During the study period, VMWs were required to test patients with the CNMCP-provided malaria RDT simultaneously, with either the Dengue Duo or Malaria/CRP RDT.

Positive CRP results (> 20 mg/L) were more common (3.2%), and VMWs followed the management algorithm and correctly referred 73% (35/48) of the CRP-positive patients. For those whom a referral was advised, 43% (15/35) attended a governmental healthcare facility, and 87% (13/15) of those were prescribed antibiotic treatment. None of these patients required onward referral to a hospital. Most patients were CRP-negative, suggesting that the majority of febrile patients presenting to VMWs (excluding those requiring urgent medical attention at the point of presentation) were appropriate for initial management in the community. Knowledge of CRP, its utility and interpretations was limited among VMWs. Further confusion arose around a positive CRP result indicating a possible bacterial infection due to the fact that in everyday Khmer language, there is no distinction between viruses, bacteria, or parasites. Nonetheless, VMWs were found to be competent in following the CRP test management algorithm. Although VMWs did not fully understand the concept and implications of CRP, competency assessment and field observations demonstrated that the majority could perform this test well, with a few having difficulties mixing the blood sample with the diluent (a step not required when using a conventional malaria RDT). A CRP RDT (within a combo-RDT or alone) that functions similarly to the CRP component of the Malaria/CRP Duo could be easily integrated into the routine practice of VMWs and could aid the management of febrile illness in the community. The cut-off for a positive CRP test result with the Malaria/CRP Duo was 20 mg/L, a threshold that emphasises sensitivity over specificity, which may be most appropriate for use by VMWs in remote Cambodian villages [38, 45, 46]. 

Despite this, the local context and their use must be considered. VMWs in Cambodia are not currently permitted to prescribe antibiotics; therefore, all positive results require onward referral to a health centre. A high proportion of these patients, despite having a positive test (> 20 mg/L), would probably have a self-limiting illness that would not require antibiotic treatment, so these patients would not benefit from referral, nor would it be a good use of limited resources. If VMWs were able to prescribe a limited number of antibiotics and/or a RDT with greater differentiation of CRP levels was used, community CRP testing might be more promising. As long as VMWs cannot provide antibiotics, CRP testing might be more effectively implemented at the health centre level [44], as has been shown in previous studies in neighbouring countries, Thailand and Vietnam [36, 47]. 

Active dengue infection was identified in 1.2% (11/915) of patients, but only two of the eleven dengue-positive (NS1- and/or IgM-positive) patients were correctly advised to attend a healthcare facility for confirmation of diagnosis and further management. An additional three patients who were positive for IgG alone were also advised of referral, which was not consistent with the management algorithm provided to VMWs, highlighting the confusion sometimes faced with interpreting the Dengue Duo results. No onward referral to a hospital was required for any of these patients. Nine dengue-positive (NS1- and/or IgM-positive) patients were provided with fever management and safety netting advice instead of referral, as would have been suggested by the management algorithm. On further discussion with the operational study team, it was suggested that some VMWs record this action if patients do not want to attend a health centre, even though the VMW understood that they should recommend a referral. This practice occurred for both combo-RDTs. A minority of VMWs may not have fully understood the testing and management process.

Despite only 11 active dengue infections being identified, VMWs stressed the importance of dengue testing. Within their communities, dengue was perceived as a frequent cause of illness. Reassurance that febrile patients were dengue-negative, especially children, was often stated as a positive outcome of the Dengue-Duo RDT deployment, highlighting the usefulness of negative and positive test results. National dengue surveillance data subsequently revealed that the study took place during a “non-peak” period, somewhat explaining the low numbers identified [48, 49]. Nevertheless, VMWs reported multiple cases in which a patient tested dengue-negative but was subsequently diagnosed with dengue fever at a private clinic and given intravenous fluid injections. These contradictions were deemed by VMWs to compromise the trust and relationships they had built in their communities over many years. The respondents reported that such discrepancies arose due to conflicting interests, as private practitioners had to convince patients that they required intravenous fluids, which ultimately served their business interests. Whether this is true or whether the private clinics simply misinterpreted an IgG-positive dengue result (indicative of previous exposure) as an active infection is unclear. Interestingly, this issue was not highlighted as problematic for the many febrile patients presenting to VMWs who currently test negative for malaria, perhaps because private clinics are not permitted to sell antimalarials in Cambodia. False negatives should also be considered, as the dengue virus NS1 antigen and IgM antibodies are detectable during different stages of infection; [50] however, the Dengue Duo RDT detects both NS1 and IgM, so it should be comparable to PCR testing [51]. Nevertheless, observations during the competency assessment and feedback from the study team identified difficulties with conducting the Dengue Duo RDT, namely collecting the correct blood sample volume for each test (100 µL for NS1 versus 10 µL for IgG/IgM). This may have led to some false negative results during deployment. Retesting negative-tested patients after 48 h who had ongoing symptoms, as indicated in the management algorithm, would have lowered the risk of missing an infection further, but unfortunately, patients rarely returned for follow-up testing. Reassuringly, during the period of the study, no deaths or adverse outcomes were reported to the operational field team. Despite the low positivity rates, alternative strategies, such as seasonal deployment (peak dengue season), may improve feasibility and aid surveillance. Usability concerns would still need to be addressed.

### Patient management

To ensure that patients testing combo-RDT-positive (CRP > 20 mg/L; Dengue NS1- and/or IgM-positive) received safe and adequate care, referral to a health centre was an integral component of the VMW management algorithm (malaria cases were managed according to national guidelines in line with existing VMW practice). VMWs stated that they appreciated the algorithm and understood its steps. Competency assessments with VMWs reassuringly suggested that most VMWs were able to refer appropriately when needed, although this was not always fully reflected during combo-RDT deployment. With sufficient training and competency-based selection, VMWs have the potential to undertake the more complex role of managing additional diseases.

Having test results available quickly (after 15 to 20 min) was much appreciated by both VMWs and patients, especially compared with the time patients otherwise had to spend travelling to health centres. VMWs were also keen to diagnose other diseases, including diabetes, tuberculosis and typhoid, somewhat aligning with the regional health priorities for primary and secondary care [52]. Earlier research with VMWs in Cambodia has shown that community members trust RDT diagnoses, even when performed by lay health workers. Additionally, the lack of tests for diseases other than malaria was a barrier to the uptake of VMW services [53]. 

### Practical concerns

With extensive experience using malaria RDTs, VMWs are good candidates for deploying other RDTs that use a similar methodology; however, not all RDTs are the same. Differences exist in the amount of blood that is needed, the steps that are needed to mix blood with diluents, and the buffers that should be added. This study demonstrated that the Malaria/CRP Duo RDT could be performed well by the majority of VMWs. The Dengue Duo RDT proved problematic with the differing amounts of blood required for the tests, along with the difficulties in obtaining the required 100 µL from a single finger prick. Previous studies have demonstrated similar difficulties with blood transfer devices for lay health workers [54, 55]. Such practical barriers to using the Dengue Duo RDT may impact VMWs’ confidence in expanding their roles due to the decreased reliability in the results and concerns with patient discomfort. This is likely to be the main reason that fewer Dengue Duo RDTs were performed compared to the Malaria/CRP RDT. The interpretation of the Dengue Duo results also proved confusing at times. While the principle is similar to other lateral flow RDTs, with a test and a control line, the Dengue Duo result needs further interpretation, as it contains two test lines (dengue IgG or IgM), which must also be considered along with the NS1 result. These factors should be considered for any new RDT that is to be rolled out to VMWs in the future, along with the amount of training required for VMWs to be competent in their use. Health centres with higher-skilled personnel may be better suited for the deployment of more complex combo-RDTs.

### Strengths and limitations

This mixed methods study broadly explored how the VMW role could be expanded using two different combo-RDTs. By incorporating the views of VMWs, health centre staff, and community members through conversations, observations and FGDs, a wide range of opinions were collated. Real-time combo-RDT usage, including result interpretations and management, and VMW consultation rates provide pragmatic insights into the potential of incorporating these tests into the routine roles of VMWs.

There were several limitations to this study, the main one being that the research was operational, so it did not include a control group of VMWs without combo-RDTs, nor did it allow for rigorous outcome tracking, and included only a small number of VMWs from the vast Cambodian VMW network. Second, researchers were present during FGDs, so social desirability bias may have affected participants’ perspectives, but this bias was somewhat mitigated through cross-checking observation notes and informal conversations. Third, competency assessments and FGDs may not have been fully representative of all VMWs. Finally, it transpired that the study took place during a “non-peak” dengue transmission period, potentially impacting the usefulness of the Dengue Duo RDT. False-negative results secondary to blood collection difficulties may have also compounded this issue.

### Implications

The primary aim of VMW role expansion is the continued uptake of VMW malaria services until malaria elimination has been achieved. Additional benefits, which would continue beyond malaria elimination, are the potential to increase the reach of primary healthcare services in rural areas, thereby reducing the burden on health centres and strengthening community-based disease surveillance through improved access to timely diagnostics and/or the provision of non-communicable disease monitoring services.

Since 2021, the Cambodian NMCP has been integrating VMWs into the healthcare system through role expansion. This involves a gradual integration of VMWs into existing community healthcare roles (Village Health Support Groups) to address other vector-borne diseases such as chikungunya, dengue, lymphatic filariasis, soil-transmitted helminths, and schistosomiasis [15]. A promising and feasible new role currently being considered for VMWs is health promotion [9]. Whilst broadening their remit to maternal and child health, and non-communicable diseases is appealing, further engagement with other departments within the Ministry of Health is needed whilst remaining mindful of the capabilities required to take on these additional roles. Given the potential conflicts with private clinics and the impact that this could have on community trust, careful consideration should be given as to how best to engage the community to mitigate this risk.

Expanded roles for community malaria workers are also being widely explored in neighbouring countries, and whilst some workers already provide additional services, i.e. basic first aid and simple fever management, others solely manage malaria [56, 57]. In Myanmar, expanding the role to allow for the management of respiratory and diarrhoeal illnesses, the detection and treatment of acute malnutrition, and tuberculosis active case finding, led to a sustained uptake of services and a decline in malaria cases [20]. Data collection, diarrhoeal illness management, health promotion, non-malarial disease symptom screening (± RDTs), and support for non-communicable diseases i.e. blood pressure and diabetes monitoring, and mental health referrals are some of the alternative interventions under consideration. Nevertheless, this role expansion requires robust training, supervision, and resource allocation to ensure diagnostic accuracy and satisfactory integration into the broader healthcare system. Long-term sustainability and equitable access must also be prioritised to ensure lasting public health benefits while maintaining VMWs’ critical role as trusted healthcare providers [56–58]. 

## Conclusions

This study demonstrated that during a short period of deploying combo-RDTs, there was increased uptake of VMW services; however, low positivity rates and the absence of actionable outcomes within the existing context of VMW services (e.g., the inability to supply antibiotics vs. the ability to supply antimalarials) suggest that other interventions may be better suited for VMW role expansion at the current time. VMWs demonstrated adequate performance with the new combo-RDTs and were able to follow simple management algorithms. The Dengue Duo RDT proved more challenging to use and interpret than the Malaria/CRP RDT. Both VMWs and community members were receptive to the concept of expanding the roles of VMWs through skills and knowledge transfer for a wider range of diseases other than malaria.

## Electronic supplementary material

Below is the link to the electronic supplementary material.

Supplementary Material 1

## Data Availability

The data are available upon request to the Mahidol Oxford Tropical Medicine Research Unit Data Access Committee (www.tropmedres.ac/units/moru-bangkok/bioethics-engagement/data-sharing), which comply with the data access policy (www.tropmedres.ac/units/moru-bangkok/bioethics-engagement/data-sharing/moru-tropical-network-policy-on-sharing-data-and-other-outputs).

## Abbreviations

CHW: Community health worker
CRP: C-reactive protein
eCRF: electronic case record form
FGD: Focus group discussion
G6PD: Glucose-6-phosphate dehydrogenase
GMS: Greater Mekong Subregion
HC: Health centre
HRP-2: Histidine-rich protein 2
IgG: Immunoglobulin G
IgM: Immunoglobulin M
CNMCP: Cambodian National Malaria Control Program
NS1: Non-structural protein 1
PCR: Polymerase chain reaction
pLDH: Plasmodium lactate dehydrogenase
RDT: Rapid diagnostic test
VMW: Village malaria worker
