# Supplementary material for: Operational evaluation of the deployment of Malaria/CRP Duo and Dengue Duo rapid diagnostic tests for the management of febrile illness by village malaria workers in rural Cambodia
Source: BMC Infect Dis. 2025 May 8;25:679. doi: 10.1186/s12879-025-11016-z (PMC12063439; doi:10.1186/s12879-025-11016-z)
Supplement: Supplementary file 1 — Supplementary Material 1 [file 12879_2025_11016_MOESM1_ESM.pdf]

# **Supplementary Appendix for "*Operational evaluation of the deployment of Malaria/CRP Duo and Dengue Duo rapid diagnostic tests for the management of febrile illness by Village Malaria Workers in rural Cambodia*"**


Appendix I. Patient management algorithm

Appendix II. Competency assessment questionnaire

Appendix III. Interrupted time series sensitivity analysis

Appendix IV. Competency assessment results

Appendix V. Graph of VMW consultation rates (total) and malaria cases in Battambang and Pailin Provinces from 2019 to 2023

Appendix VI. Sensitivity and specificity of Malaria/CRP and Dengue Duo RDTs

## Appendix I. Patient management algorithm

*Malaria/CRP Duo*

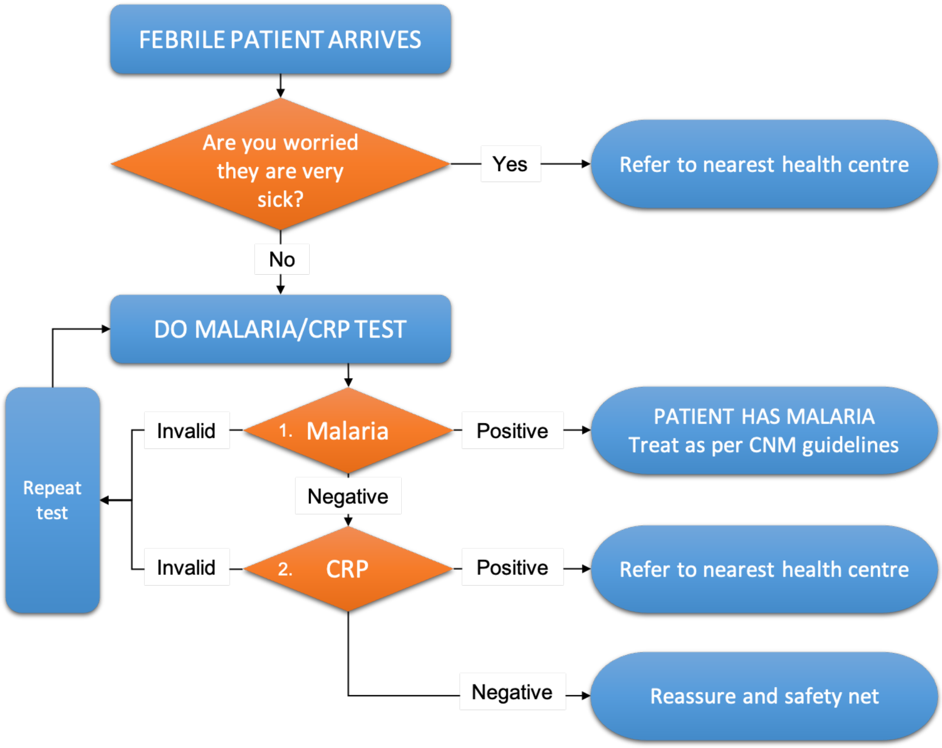


*Dengue Duo*

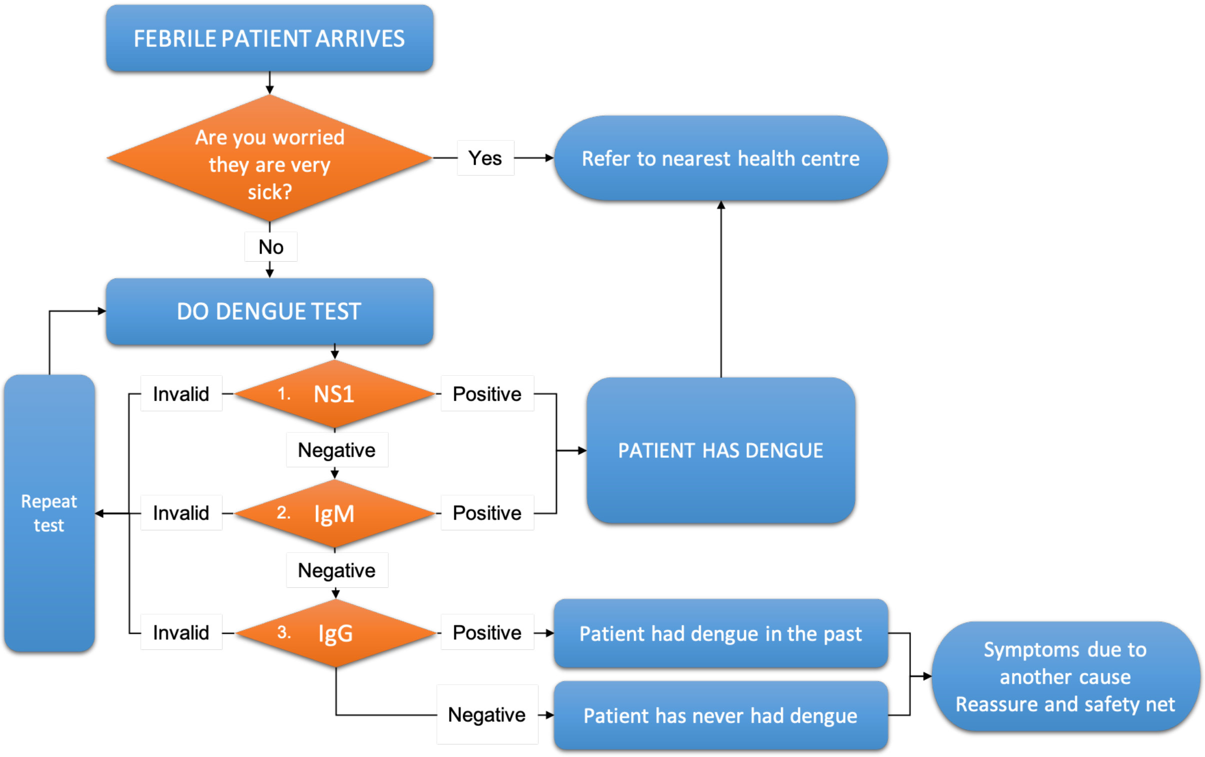

## Appendix II. Competency assessment questionnaire

### Malaria/CRP checklist

| No. | Description | Score | | | Assessor's notes (please note the details or circumstances of the error) |
|---|---|---|---|---|---|
| | | 0 | 1 | 2 | |
| **Comprehension** – Ask the participants the following questions | | | | | |
| 1 | What is malaria? | | | | |
| 2 | What are the symptoms of malaria? | | | | |
| 3 | What is CRP? | | | | |
| 4 | What does a high CRP level mean? | | | | |
| **Malaria test** | | | | | |
| 5 | Ask participants to perform or explain the Malaria RDT procedure. | | | | *Explained / performed* |
| **CRP test** | | | | | |
| 6 | Ask participants to perform or explain the CRP RDT procedure. | | | | *Explained / performed* |
| **Reading the results and understanding the treatment algorithm** | | | | | |
| 7 | Ask the participants when they read the test results. | | | | |
| 8 | Malaria test. What is the result? What action should be taken? 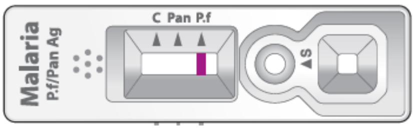 | | | | |
| 9 | Malaria test. What is the result? What action should be taken? 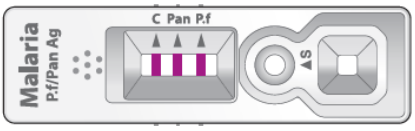 | | | | |
| 10 | CRP test. What is the result? What action should be taken? 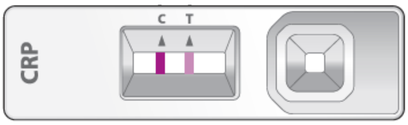 | | | | |
| 11 | CRP test. What is the result? What action should be taken? 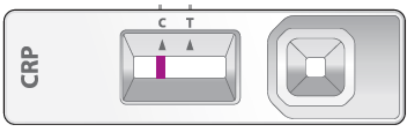 | | | | |

# SCORING

**Comprehension**
No. 1 - *Malaria is an infection* ***spread by mosquitos***
No. 2 - ***Fever*** *and sometimes more serious illness/severe malaria*
No. 3 - *CRP is something the body makes in response to* ***infection***
No. 4 - *The infection is more likely to require* ***antibiotics***

1 point for each correct answer (must include keyword in bold)

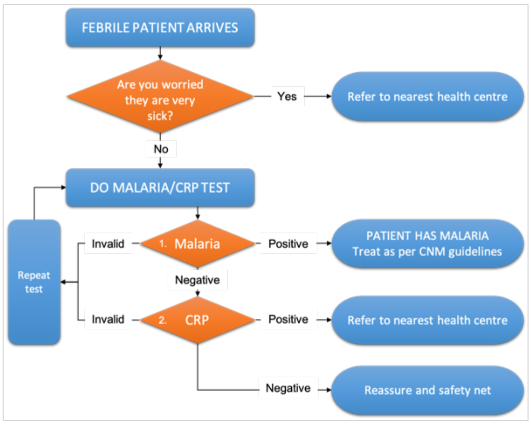


**Malaria test**
Steps are as follows:
a. *Wipe patient's finger with an alcohol wipe and prick with a lancet.*
b. *Transfer the blood (5µl) to the specimen well (circle well) of the test device*
c. *Add 3 drops of buffer (90µl) to the square well of the test device*

No. 5
0 points if one or more steps are not performed
1 point if one or more steps are performed incorrectly
2 points if all steps are performed correctly and in order

**CRP test**
Steps are as follows:
a. *Collect the blood with the Ezi tube, filling it to the black line (10µl)*
b. *Add the blood to the assay diluent. Dispose the Ezi tube*
c. *Use the dropper (100µl) to mix the specimen by pressing and releasing the bulb 6-8 times*
d. *Collect all specimen using the dropper and add all to the sample well*

No. 6
0 points if one or more steps are not performed
1 point if one or more steps are performed incorrectly
2 points if all steps are performed correctly and in order

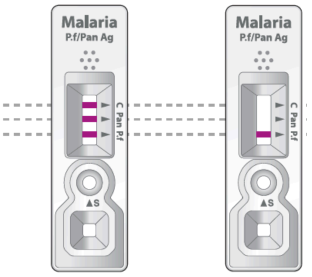


Pf (*Plasmodium falciparum*) Positive or mixed Positive of Pf and Pv, Po, Pm

Invalid

**Reading the results and understanding the treatment algorithm**
No. 7
0 points for reading results before 15 or after 20 minutes
2 points for reading results between 15 and 20 minutes

No. 8 - Test is invalid. Test should be repeated
No. 9 - Test is positive for (*P. falciparum or mixed*) malaria. Patient should be treated according to CNM guidelines
No. 10 - CRP level is lower than 20mg/L. Patient should be reassured and be given a safety net
No. 11 - CRP level is higher than CRP > 20 mg/L. Patient should be referred to the nearest health center

0 points if the test result is read incorrectly
1 point if the test result is read correctly
2 points if the test result is read correctly and appropriate action is taken according to the treatment algorithm

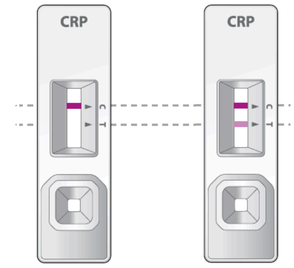


CRP level is lower than 20mg/L

CRP level is above 20mg/L

*All Malaria/CRP RDT images are adapted from the Standard Q Malaria/CRP Duo product insert*

# Dengue Duo checklist

| No. | Description | Score | | | Assessor's notes (please note the details or circumstances of the error) |
|---|---|---|---|---|---|
| | | 0 | 1 | 2 | |
| **Comprehension** – Ask the participants the following questions | | | | | |
| 1 | What is dengue? | | | | |
| 2 | What are the symptoms of dengue? | | | | |
| **NS1 Test** | | | | | |
| 3 | Ask participants to explain or perform the NS1 procedure. | | | | *Explained / performed* |
| **Antibody test** | | | | | |
| 4 | Ask participants to explain or perform the antibody procedure. | | | | *Explained / performed* |
| **Reading the results and understanding the treatment algorithm** | | | | | |
| 5 | Ask the participants when they read the test results. | | | | |
| 6 | NS1 test. What is the result? What action should be taken? 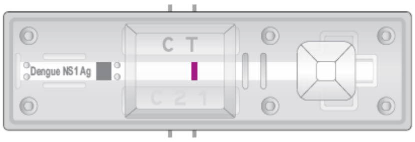 | | | | |
| 7 | NS1 test. What is the result? What action should be taken? 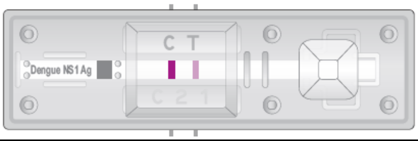 | | | | |
| 8 | Antigen test. What is the result? What action should be taken? 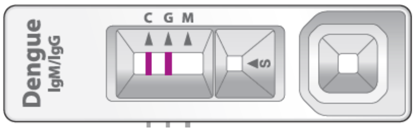 | | | | |
| 9 | Antigen test. What is the result? What action should be taken? 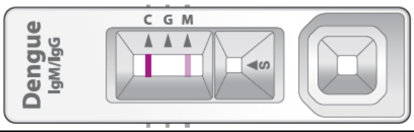 | | | | |
| 10 | Antigen test. What is the result? What action should be taken? 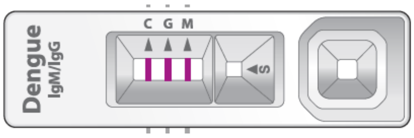 | | | | |

## SCORING

**Comprehension**

<u>No. 1</u> – *Dengue is an infection **spread by mosquitos***

<u>No. 2</u> – ***Fever/febrile illness**, with muscle/joint/bone pain.*

2 points for each correct answer (must include keyword in bold)

**NS1 test**

Steps are as follows:

a. *Wipe patient's finger with an alcohol wipe and prick with a lancet*

b. *Collect the blood using the sample device, filling it to the black line (100µl)*

c. *Transfer the blood to the NS1 test device*

<u>No. 3</u>

0 points if one or more steps are not performed

1 point if one or more steps are performed incorrectly

2 points if all steps are performed correctly and in order

**Antibody test**

Steps are as follows:

a. *Collect the blood using the Ezi tube, filling it to the black line (10µl)*

b. *Transfer the blood to the specimen well (smaller well) of the test device*

c. *Add 3 drops of buffer (90µl) to the square well of the test device*

<u>No. 4</u>

0 points if one or more steps are not performed

1 point if one or more steps are performed incorrectly

2 points if all steps are performed correctly and in order

**Reading the results and understanding the treatment algorithm**

<u>No. 5</u>

0 points for reading results before 15 or after 20 minutes

2 points for reading results between 15 and 20 minutes

<u>No. 6</u> - Test is invalid. **Repeat** test.

<u>No. 7</u> - Test is NS1 positive. **Refer patient**; dengue may be the cause of the fever

<u>No. 8</u> - Test is IgG positive. **Reassure and safety-net**; patient may have had dengue in the past but unlikely to be the cause of the fever today

<u>No. 9</u> - Test is IgM positive. **Refer patient**; dengue may be the cause of the fever

<u>No. 10</u> - Test is both IgM and IgG positive. **Refer patient**; dengue may be the cause of the fever

0 points if the test result is read incorrectly

1 point if the test result is read correctly

2 points if the test result is read correctly and appropriate action is taken according to the treatment algorithm

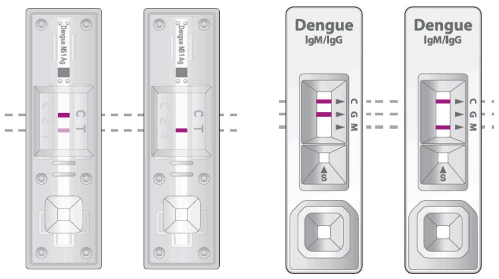


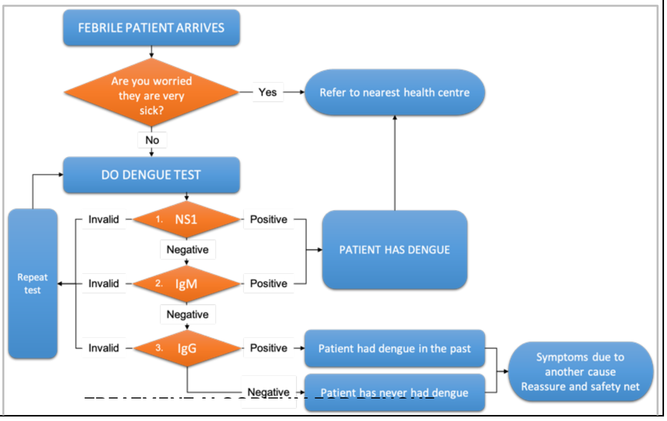


*All Dengue Duo RDT images are adapted from the Standard Q Dengue Duo product insert*

## Appendix III. Interrupted time series sensitivity analysis using Prais-Winsten regression

The models reported in the manuscript do not include seasonality as a covariate. As a sensitivity analysis, alternate models were fitted with the following:

1. Adjustment for seasonality with a categorical month indicator as a covariate.
2. Adjustment for seasonality with a categorical wet season indicator as a covariate.

For sensitivity analyses of the interrupted time series analysis (Table 2- main text), estimates and confidence intervals are similar when adjusting for seasonality with a categorical month indicator (sensitivity analysis model 1) and when adjusting for seasonality with a categorical wet season indicator (sensitivity analysis model 2).

| | **Pre-intervention trend [a] (consultations per month), (95% CI); p value** | **Step change at RDT introduction (consultations per month), 95% CI); p value** | **Post-intervention trend (consultations per month), 95% CI); p value** |
|---|---|---|---|
| **Original model** | | | |
| Study VMWs (combo-RDTs) *n = 84* | -0.18 (-0.40 to 0.04); p = 0.11 | 4.4 (1.5 to 7.4); p <0.01 | -0.02 (-0.43 to 0.39); p = 0.92 |
| **Sensitivity analysis model 1** | | | |
| Study VMWs (combo-RDTs) *n = 84* | -0.19 (-0.49 to 0.10); p = 0.22 | 4.7 (0.4 to 8.9); p = 0.05 | -0.04 (-0.59 to 0.52); p = 0.90 |
| **Sensitivity analysis model 2** | | | |
| Study VMWs (combo-RDTs) *n = 84* | -0.18 (-0.41 to 0.04); p = 0.12 | 4.5 (1.4 to 7.5); p <0.01 | -0.02 (-0.44 to 0.39); p = 0.91 |

[a] Pre-intervention trend in the study area was derived from VMW data collected in the 18 months prior to deployment.
p-values refer to a null effect i.e. trend: slope = 0 and step-change = 0
VMWs – Village malaria workers, CI – confidence intervals

## Appendix IV. Competency assessment results

***A. Malaria/CRP Duo***

| | Score | | Q1 | Q2 | Q3 | Q4 | Q5 | Q6 | Q7 | Q8 | Q9 | Q10 | Q11 | Error made |
|---|---|---|---|---|---|---|---|---|---|---|---|---|---|---|
| VMW 1 | 16 | | 1 | 1 | 0 | 1 | 2 | 2 | 1 | 2 | 2 | 2 | 2 | CRP |
| VMW 2 | 15 | | 1 | 1 | 0 | 1 | 2 | 1 | 1 | 2 | 2 | 2 | 2 | CRP; mix |
| VMW 3 | 14 | | 1 | 1 | 1 | 0 | 2 | 0 | 1 | 2 | 2 | 2 | 2 | bacteria; material; mix |
| VMW 4 | 13 | | 1 | 1 | 0 | 0 | 2 | 0 | 1 | 2 | 2 | 2 | 2 | CRP; bacteria; material; mix |
| VMW 5 | 16 | | 1 | 1 | 0 | 1 | 2 | 2 | 1 | 2 | 2 | 2 | 2 | CRP |
| VMW 6 | 16 | | 1 | 1 | 1 | 0 | 2 | 2 | 1 | 2 | 2 | 2 | 2 | bacteria |
| VMW 7 | 16 | | 1 | 1 | 0 | 1 | 2 | 2 | 1 | 2 | 2 | 2 | 2 | CRP |
| VMW 8 | 16 | | 1 | 1 | 1 | 0 | 2 | 2 | 1 | 2 | 2 | 2 | 2 | bacteria |
| | *Maximum score* | 17 | 1 | 1 | 1 | 1 | 2 | 2 | 1 | 2 | 2 | 2 | 2 | |
| | *Median score* | 16 | | | | | | | | | | | | |

Q1 - Q2: malaria knowledge; Q3 - Q4: CRP knowledge, Q5 practical malaria test performance; Q6 practical CRP test performance; Q7 - Q11 test result reading.
Errors made consisted of (bacteria) not answering bacteria/bacterial infection, (CRP) not knowing what CRP is, (material) using the wrong material for the test, and (mix) making a mistake while mixing blood with buffer.

***B. Dengue Duo***

| | Score | | Q1 | Q2 | Q3 | Q4 | Q5 | Q6 | Q7 | Q8 | Q9 | Q10 | Error made |
|---|---|---|---|---|---|---|---|---|---|---|---|---|---|
| VMW 9 | 11 | | 1 | 1 | 1 | 2 | 1 | 1 | 0 | 2 | 0 | 2 | Blood; interpretation; action |
| VMW 10 | 12 | | 1 | 1 | 1 | 0 | 1 | 2 | 2 | 0 | 2 | 2 | Blood; order; test; interpretation |
| VMW 11 | 16 | | 1 | 1 | 1 | 2 | 1 | 2 | 2 | 2 | 2 | 2 | Blood |
| VMW 12 | 16 | | 1 | 1 | 1 | 2 | 1 | 2 | 2 | 2 | 2 | 2 | Blood |
| VWM 13 | 16 | | 1 | 1 | 2 | 1 | 1 | 2 | 2 | 2 | 2 | 2 | Blood |
| VMW 14 | 15 | | 1 | 1 | 0 | 2 | 1 | 2 | 2 | 2 | 2 | 2 | Blood |
| VMW 15 | 17 | | 1 | 1 | 2 | 2 | 1 | 2 | 2 | 2 | 2 | 2 | |
| VMW 16 | 16 | | 1 | 1 | 1 | 2 | 1 | 2 | 2 | 2 | 2 | 2 | Blood |
| | *Maximum score* | 17 | 1 | 1 | 2 | 2 | 1 | 2 | 2 | 2 | 2 | 2 | |
| | *Median score* | 16 | | | | | | | | | | | |

Q1 - Q2: dengue knowledge; Q3 practical NS1 test performance; Q4 practical IgM/IgG test performance; Q5 - Q10 test result reading.
Errors made consisted of (action) incorrect action taken, (blood) too little blood used, (interpretation) incorrect test interpretation, (order) steps taken in incorrect order, and (test) wrong test used

**Appendix V. VMW consultation rates (total) and malaria cases in Battambang and Pailin Provinces from 2019 to 2023**

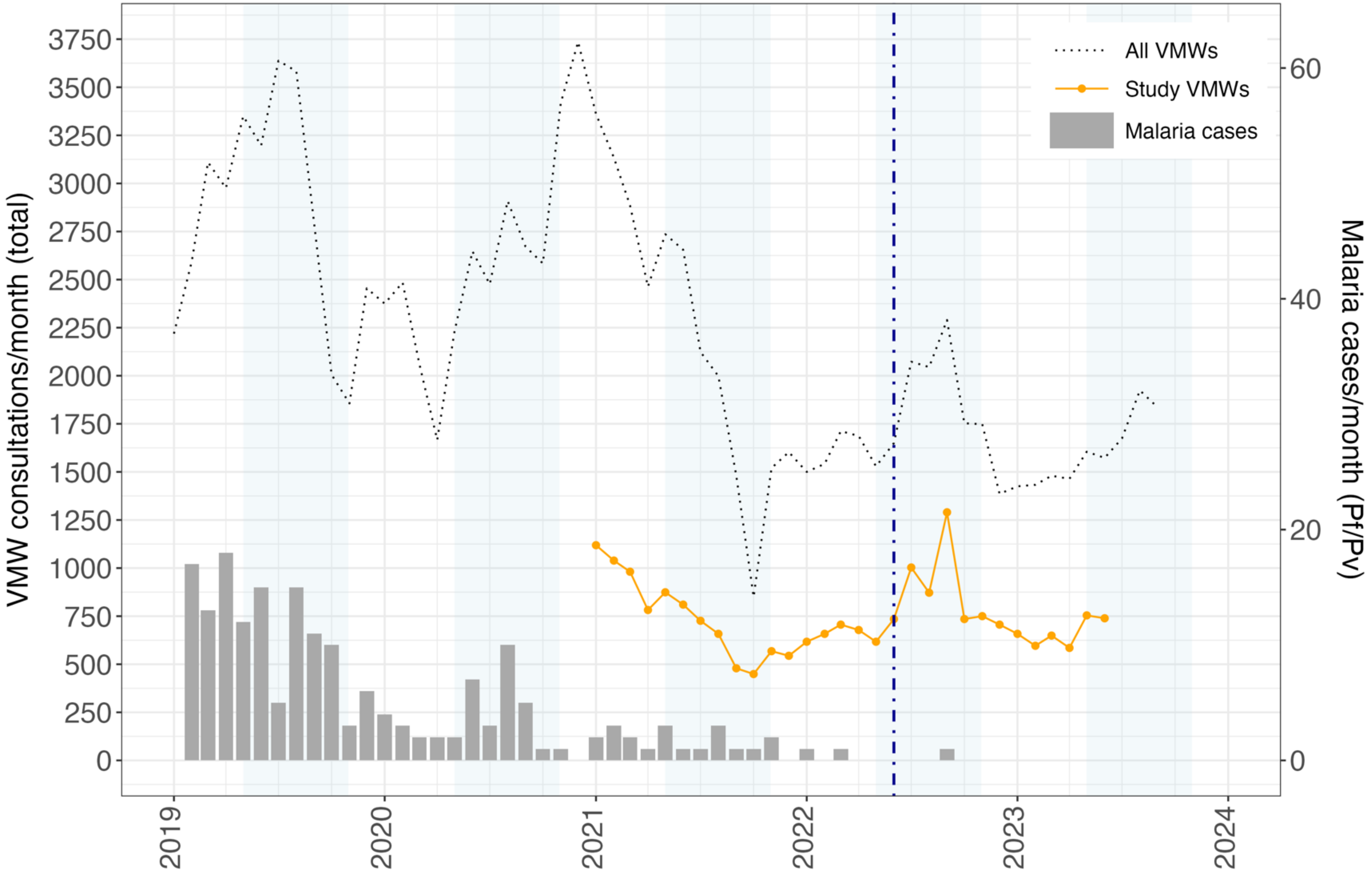


Monthly VMW consultations for the study VMWs (orange line) and all VMWs in Battambang/Pailin Provinces (black dotted line). The vertical blue line denotes the time when the Dengue Duo and Malaria/CRP RDTs (combo-RDTs) were deployed. The bar chart depicts the total monthly malaria cases (Pf/Pv) in Battambang/Pailin Provinces. The blue panels represent the timing of the rainy season.

## Appendix VI. Sensitivity and specificity of Malaria/CRP and Dengue Duo RDTs

The sensitivity and specificity metrics for both the Malaria/CRP and Dengue Duo RDTs have been adapted from the manufacturer product information.

Standard Q Dengue Duo

| | Reference | Sensitivity | Specificity |
|---|---|---|---|
| NS1 | RT-PCR | 92.9% (184/198) | 98.7% (222/225) |
| IgM | ELISA | 97.5% (77/79) | 96.6% (346/358) |
| IgG | ELISA | 97.2% (140/144) | 96.2% (282/293) |

*Reference: Internal evaluation*

Standard Q Malaria/CRP Duo

| | Reference | Sensitivity | Specificity |
|---|---|---|---|
| P.f | Microscopy | 99.58% (476/478) | 100% (1000/1000) |
| P.v, P.m and P.o confirmed specimen on Pan | Microscopy | 100% (129/129) | |
| CRP | Immunoturbidimetric | 87.5% (21/24) | 100% (50/50) |

*Reference: Internal evaluation*
